# Comparative Clinical Outcomes of Nusinersen and Gene Therapy in Spinal Muscular Atrophy Type 1

**DOI:** 10.1001/jamanetworkopen.2025.36348

**Published:** 2025-10-08

**Authors:** Juliette Ropars, Claude Cances, Rocio Garcia-Uzquiano, Marta Gomez-Garcia de la Banda, Christine Barnerias, Frédérique Audic, Julien Durigneux, Cécile Halbert, Lionelle Nkam, Vincent Laugel, Caroline Espil, Ulrike Walther-Louvier, Jean-Baptiste Davion, Arnaud Isapof, Laure Le Goff, Isabelle Desguerre, Susana Quijano-Roy, Lamiae Grimaldi

**Affiliations:** 1 Reference Center for Neuromuscular Disorders, Pediatric Department, CHU Brest, Brest, France; 2 Laboratoire de Traitement de l’Information Médicale (LaTIM) INSERM UMR 1101, Brest, France; 3 Unité de Recherche Clinique Assistance Publique–Hôpitaux de Paris, Université Paris-Saclay, Direction de la Recherche Clinique Assistance Publique Hôpitaux de Paris, Paris, France; 4 Reference Center for Neuromuscular Disorders, Pediatric Clinical Research Unit/Pediatric Multi-Thematic Module CIC 1436, Neuropediatric Department, Toulouse University Hospital, Toulouse, France; 5 Reference Center for Neuromuscular Disorders, Neuropediatric Department, Hôpital Raymond Poincaré, Garches, France; 6 Reference Center for Neuromuscular Disorders, Neuropediatric Department, Hôpital Necker Enfants Malades, Paris, France; 7 Reference Center for Neuromuscular Disorders, Neuropediatric Department, Hôpital de la Timone, Assistance Publique–Hôpitaux de Marseille, Marseille, France; 8 Reference Center for Neuromuscular Disorders, Neuropediatric Department, Angers University Hospital, Angers, France; 9 Reference Center for Neuromuscular Disorders, Neuropediatric Department, Strasbourg University Hospital, Strasbourg, France; 10 Reference Center for Neuromuscular Disorders, Neuropediatric Department, Bordeaux University Hospital, Bordeaux, France; 11 Reference Center for Neuromuscular Disorders, Neuropediatric Department, Montpellier University Hospital, Montpellier, France; 12 Reference Center for Neuromuscular Disorders, Department of Neurology, Lille University Hospital, Lille, France; 13 Reference Center for Neuromuscular Disorders, Neuropediatric Department, Hôpital Trousseau, Assistance Publique–Hôpitaux de Paris, Paris, France; 14 Reference Center for Neuromuscular Disorders, Department of Pediatric Physical Medicine and Rehabilitation, Hôpital Mère Enfant, Lyon University Hospital, Bron, France; 15 Neuromyogen Institute, Université de Lyon, Lyon, France; 16 Laboratoire END-ICAP-UMR 1179 (INSERM/UVSQ), Handicap Neuromusculaire: Physiopathologie, Biothérapie et Pharmacologie Appliquées, Montigny-le-Bretonneau, France; 17 Team Anti-Infective Evasion and Pharmacoepidemiology, INSERM UMR1018, Faculty of Medicine Simone Veil, University Versailles Saint-Quentin-en-Yvelines-Paris Saclay U, Montigny-Le-Bretonneux, France

## Abstract

**Importance:**

Therapeutic advances have transformed the prognosis of spinal muscular atrophy (SMA). Given the lifelong implications of these innovative therapies, comparative data on their efficacy are urgently required.

**Objective:**

To compare clinical outcomes of nusinersen and onasemnogene abeparvovec gene therapy as first-line treatments in children with SMA type 1 (SMA1).

**Design, Setting, and Participants:**

This comparative effectiveness study used data from the French National SMA Registry from September 2016 to July 2024. The follow-up period started at treatment initiation and continued until July 22, 2024, or death. Children with genetically confirmed SMA1 (types a, b, or c) treated within 6 months of diagnosis with either nusinersen or gene therapy as first-line therapy and followed up for at least 24 months were included. Matching criteria included age, baseline score on the Children’s Hospital of Philadelphia Infant Test of Neuromuscular Disorders, and respiratory and nutritional status at treatment initiation.

**Exposure:**

First-line treatment with either nusinersen or gene therapy.

**Main Outcomes and Measures:**

Outcomes included respiratory and nutritional support needs, motor function, and unsatisfactory clinical response (UCR)— a composite of death, treatment switch (or, for gene therapy, addition) due to inadequate response, initiation of feeding support, and/or failure to achieve independent sitting.

**Results:**

Among 1366 patients enrolled in the registry, 309 were diagnosed with SMA1. Twenty-four children in 12 matched pairs met inclusion criteria (14 [58%] male; mean [SD] age at treatment initiation, 6.1 [3.0] months [range, 2.3-11.9 months]). Three patients (1 receiving gene therapy [8%], 2 receiving nusinersen [17%]) died within the first year of treatment. At 2 years posttreatment, 1 of the 11 surviving patients treated with gene therapy (9%) required nutritional support vs 5 of 10 (50%) treated with nusinersen, and nocturnal ventilation was required in 5 of 11 (45%) receiving gene therapy vs 8 of 10 (80%) receiving nusinersen. Motor outcomes were comparable between groups (mean [SE] intrapair difference in CHOP-INTEND score evolution, −1.69 [1.24] points; *P* = .17). UCR occurred in 8 of 12 patients (67%) receiving nusinersen and 3 of 12 (25%) receiving gene therapy.

**Conclusions and Relevance:**

In this comparative effectiveness study of children with SMA1, gene therapy was associated with lower incidence of UCR and fewer supportive care needs vs nusinersen. These exploratory findings warrant confirmation in larger studies.

## Key Points

**Question:**

What are the differences in clinical outcomes in children with spinal muscular atrophy (SMA) type 1 receiving first-line gene therapy vs nusinersen?

**Findings:**

In this comparative effectiveness study of 24 children in 12 matched pairs using data from the French National SMA Registry, children treated with first-line gene therapy experienced lower incidence of unsatisfactory clinical response and required less respiratory and nutritional support over time compared with those treated with first-line nusinersen, with similar motor outcomes.

**Meaning:**

The findings support consideration of gene therapy as a potentially preferable treatment option in SMA type 1 and may offer evidence to guide treatment decisions.

## Teaser

This comparative effectiveness study examines respiratory and nutritional support, motor function, and survival outcomes in a matched cohort of French children receiving nusinersen vs gene therapy as first-line treatment for spinal muscular atrophy type 1.

## Introduction

Spinal muscular atrophy (SMA) was the leading inherited cause of childhood death until 2016, when 3 disease-modifying treatments (DMTs) became available^1^: the antisense oligonucleotide nusinersen, gene replacement therapy using onasemnogene abeparvovec, and the small molecule-splicing modifier risdiplam. These drugs are designed to compensate for the lack of survival motor neuron (SMN) protein due to a homozygous defect in the *SMN1* gene.^2^ The clinical severity of SMA is heterogenous and mainly related to varying copy numbers of the paralogous *SMN2* gene, as high *SMN2* copy numbers are generally associated with a mild phenotype.^3^ The natural history of SMA type 1 (SMA1, most often associated with two *SMN2* copies), the most common phenotype, is characterized by onset during the first 6 months of life and severe muscle weakness, bulbar dysfunction, and respiratory insufficiency leading to premature death.

As an antisense oligonucleotide, nusinersen modifies *SMN2* splicing via intronic silencer N1 inhibition. Gene therapy delivers a complementary DNA sequence encoding functional SMN protein via an adeno-associated virus serotype 9 vector and is administered intravenously as a one-off treatment. Clinical trial data have shown both treatments improve motor function and survival without the need for permanent ventilation in individuals with SMA1.^4,5,6,7,8^ Observational studies further supported the effectiveness and safety of the treatments but highlighted variations in outcomes based on symptom onset and treatment timing.^9,10,11,12,13^ Concerns remain regarding the impact of nusinersen and gene therapy on bulbar and respiratory function.^9,11^

An increasing number of countries have introduced SMA newborn screening to facilitate timely treatment interventions, particularly for individuals at an increased risk of early disease onset.^14,15,16^ However, evidence for choosing which drug to administer remains limited. To our knowledge, the efficacy and safety profiles of DMTs have not been compared in head-to-head trials. Owing to differences in baseline characteristics between populations, indirect treatment comparisons have yielded low-quality evidence.^17^

Observational disease registries offer a pragmatic approach to compare effectiveness across therapies. In France, the Haute Autorité de Santé requested the implementation of a national SMA registry to improve knowledge of the disease’s epidemiology and natural history and to support clinical drug utilization studies, including potential comparative analyses.^18^

This study aimed to compare clinical outcomes of nusinersen and gene therapy in children with SMA1 using matched cohorts from the French National SMA Registry. Motor, respiratory, and bulbar outcomes were evaluated to assess treatment results across key functional domains with impact on health, handicap, and burden issues.

## Methods

### Study Design and Data Source Population

This comparative effectiveness study was conducted following the International Society for Pharmacoeconomics and Outcomes Research (ISPOR) reporting guideline and used data from the French National SMA Registry, which includes longitudinal clinical information on all patients with genetically confirmed 5q SMA managed since September 2016 across 64 neuromuscular centers within the French Neuromuscular Network (FILNEMUS).^18^ The registry collects standardized historical prospective data on motor, respiratory, and nutritional function, treatments, and outcomes. In France, only designated specialists can prescribe DMTs. Treatment decisions for newly diagnosed patients and for any therapy switch or add-on therapies require validation by a national multidisciplinary expert committee, based on standardized criteria such as documented motor regression, persistent bulbar dysfunction, or treatment administration challenges (eg, due to severe scoliosis or spinal hardware).^18^ This study was approved for data processing by the Assistance Publique–Hôpitaux de Paris under the European Union General Data Protection Regulation and the French Data Protection Act and was exempted from research ethics committee review by French law (Loi Jardé) as it analyzed existing data from the French National SMA Registry without any additional procedures. Participants (or their parents or legal guardians, if minors) provided written informed consent for inclusion in the registry and for the use of their health data in this study. The French National SMA Registry protocol is registered with the Health Data Hub.

### Study Population and Matching

We included children with symptomatic SMA1 who received onasemnogene abeparvovec gene therapy or nusinersen as a first-line DMT and met the following criteria at treatment initiation: had never achieved independent sitting; had 2 or 3 *SMN2* copies; were aged less than 9 months for SMA types 1a (no head control) and 1b (head control but unable to sit) or less than 12 months for type 1c (able to sit with support); were naive users of gene therapy or nusinersen at treatment initiation; initiated treatment within 6 months of diagnosis; had at least 24 months of follow-up; and were not treated as part of a clinical trial.

Exact 1:1 matching was performed on key baseline characteristics likely to influence both treatment decisions and outcomes^9^: age at treatment initiation (≤30 [±7] days, >30 to ≤90 [±15] days, or >90 [±30] days), motor function measured using Children’s Hospital of Philadelphia Infant Test of Neuromuscular Disorders (CHOP INTEND)^19^ scores (<30 [±3] or ≥30 [±5] points on a scale of 0-64, with higher scores indicating better function), and feeding and ventilatory status at treatment initiation. When multiple candidates met the matching criteria, we randomly selected 1 of the candidates.

This matching approach was chosen over propensity score methods to preserve clinical similarity in small samples.^20,21,22^ Pairwise matching characteristics are provided in eTable 1 in Supplement 1.

### Data Collection and Outcomes

The index date was the date of the first nusinersen or gene therapy administration. The follow-up period, during which outcomes were evaluated, lasted until the end of the study period (July 22, 2024), last clinical assessment, or death. Collection of data followed routine care and was previously detailed.^18^

Outcomes included survival, sustained respiratory support (either invasive or noninvasive), sustained feeding support if maintained for 31 or more consecutive days (any support vs no support), CHOP INTEND scores, and achievement of motor abilities (World Health Organization–based^23^ plus early abilities relevant to SMA1: head control and dependent or independent sitting, standing, and walking). These were assessed at variable time points across patients. We also defined a composite outcome labeled *unsatisfactory clinical response* (UCR), capturing clinically meaningful shortfalls in therapeutic outcomes. UCR was defined as death, treatment switch (or add-on, for gene therapy) due to inadequate response, initiation of nutritional support maintained for 31 or more consecutive days, and/or failure to achieve independent sitting. Ventilatory support was excluded from the UCR outcome due to heterogeneity in its indication (eg, prophylactic vs therapeutic), which was not uniformly documented in the registry. This composite outcome reflects the multidimensional nature of response in SMA1 and aligns with the study’s exploratory comparative design. Detailed definitions, study variables, and the study protocol are provided in the eMethods in Supplement 1.

### Statistical Analysis

We performed an exploratory comparative analysis using all matched pairs of children from the registry, with no prespecified primary outcome or sample size calculation. Continuous data for each population (nusinersen vs gene therapy) were summarized using descriptive statistics, including mean (SD) and median (range and IQR) values. Categorical data were summarized as the total number of patients in each category. Comparative frequencies were expressed as percentages of the total. A linear mixed-effects model was used to analyze the evolution of the change in CHOP INTEND scores over time from the index date, stratified by first-line therapy. The model accounted for nested random slopes for patient identifiers and matched pairs. Two specifications of the time variable were evaluated: number of months since treatment initiation and log (1 + months since treatment initiation), the latter accounting for potential nonlinear trends in CHOP INTEND score changes over time.

Kaplan-Meier estimates were used for time-to-event analyses of right-censored outcomes, including the need for feeding or respiratory support interventions and achievement of motor milestones. Given the small sample size and limited number of events, no formal statistical comparison between treatment groups was planned, and survival curves were presented without CIs.

Conditional logistic regression stratified by matched pairs was used to assess the association between UCR and first-line therapy. We chose to model UCR as a binary outcome rather than using a time-to-event approach, given the limited number of events and matched pairs, the heterogeneity and variable timing of individual events, and the clinical relevance of event occurrence rather than timing in this context.

Given the small number of matched pairs, we also performed a sensitivity analysis using the exact McNemar test for the comparison of UCR between groups. For within-pair comparisons of patient outcomes, analyses were restricted to the minimum follow-up time of each pair, except in cases where 1 patient had died, to avoid bias from unequal exposure to risk and ensure comparable follow-up periods.

Statistical significance was conventionally defined as a 2-sided *P* value less than .05. However, given the small sample size and the exploratory nature of the study, we emphasized effect sizes and 95% CIs over binary significance testing, in line with current recommendations for transparent clinical research reporting.^24,25^ All statistical analyses were performed using R, version 3.4.4 (R Project for Statistical Computing).

## Results

### Participants

Since its launch in January 2020, the French National SMA Registry had enrolled 1366 patients, including 309 diagnosed with SMA1. Among them, 88 met the inclusion criteria: 43 (49%) and 45 (51%) received gene therapy and nusinersen, respectively, as first-line therapy. Twelve children treated with gene therapy were successfully matched with 12 children treated with nusinersen based on baseline characteristics (Table). Details on selection and unmatched characteristics are in eFigure 1 and eTable 2 in Supplement 1.

**Table.** Characteristics of Matched Patients at Index Date According to First-Line Therapy

Characteristic	Matched group^a^	
	Gene therapy (n = 12)	Nusinersen (n = 12)
Sex		
Female	5 (42)	5 (42)
Male	7 (58)	7 (58)
Age at treatment initiation, mo^b^		
Mean (SD)	6.1 (3.0)	6.1 (3.0)
Median (IQR) [range]	4.6 (4.2-8.7) [2.3-11.9]	4.7 (3.9-8.8) [2.8-11.1]
Type of SMA^c^		
1a	1 (8)	1 (8)
1b	7 (58)	7 (58)
1c	4 (33)	4 (33)
*SMN2* copies, No.		
2	10 (83)	9 (75)
3	2 (17)	3 (25)
Age at symptom onset, mo		
Mean (SD)	3.1 (2.2)	2.8 (2.2)
Median (IQR) [range]	2.2 (1.7-4.6) [0-6.6]	2.2 (1.1-4.2) [0-6.1]
Age at genetic diagnosis, mo		
Mean (SD)	5.2 (2.9)	5.4 (3.0)
Median (IQR) [range]	4.4 (3.3-8.1) [1.2-10.8]	4.4 (3.0-6.9) [2.2-10.9]
Weight at treatment initiation, kg		
Mean (SD)	6.8 (1.7)	6.4 (1.2)
Median (IQR) [range]	6.4 (5.2-8.4) [4.3-9.4]	6.4 (5.4-7.1) [4.9-8.8]
Ventilatory support of any type at treatment initiation^b^	12 (100)	12 (100)
Feeding support at treatment initiation		
Oral feeding only^b^	11 (92)	11 (92)
Feeding support	1 (8)	1 (8)
CHOP INTEND score at treatment initiation^b^^,^^d^		
Mean (SD)	26.1 (6.1)	25.2 (6.5)
Median (IQR) [range]	27.0 (22.5-29.0) [15.0-36.0]	24.5 (20.8-27.2) [15.0-40.0]
Maximal motor ability at treatment initiation		
No capacity	5 (42)	4 (33)
Head control for >3 s	6 (50)	7 (58)
Sitting with support	1 (8)	1 (8)
Acquisition age at maximal motor ability before treatment initiation, mo		
Mean (SD)	5.9 (3.0)	5.9 (3.0)
Median (IQR) [range]	4.5 (4.1-8.4) [1.6-11.4]	4.7 (3.5-7.7) [2.8-11.1]
Follow-up time from treatment initiation to end of study period, last assessment, or death, mean (SD), mo	39.9 (17.0)	61.8 (26.1.0)

Abbreviations: CHOP INTEND, Children’s Hospital of Philadelphia Infant Test of Neuromuscular Disorders; SMA, spinal muscular atrophy.

^a^ Data are presented as number (percentage) of patients unless otherwise indicated.

^b^ Baseline characteristic used for the 1:1 matching process between the patient cohorts.

^c^ Types of SMA: 1a, no head control; 1b, head control but no sitting; 1c, sitting with support but never independently.

^d^ Score range, 0-64, with higher scores indicating better function.

Matched cohorts showed no significant differences in sex distribution (5 children [42%] in each group were female and 7 [58%] were male) or SMA subtype. The mean (SD) age at treatment initiation was 6.1 (3.0) months in both groups (total range, 2.3-11.9 months). At baseline, no patients required mechanical ventilation; 1 patient (8%) in each group required feeding support. Most patients (10 [83%] in each group) had a CHOP INTEND score lower than 30.

### Follow-Up

No missing data were reported for the motor, respiratory, or bulbar function assessments during the observation period for any patient. Three patients (1 [8%] in the gene therapy group, 2 [17%] in the nusinersen group) died from respiratory complications within 12 months, all of whom had SMA1a, 2 *SMN2* copies, and a CHOP-INTEND score less than 30 at baseline. None of the deaths were attributed to DMT-related adverse effects.

Mean follow-up was longer in the nusinersen group than in the gene therapy group, reflecting the earlier availability of nusinersen in France. Two of the 11 surviving patients treated with gene therapy (18%) received an add-on therapy because of insufficient motor improvement: 1 received risdiplam 3 years after gene therapy and the other received nusinersen 2 years after gene therapy. In the nusinersen group, 5 of the 10 surviving patients (50%) switched to risdiplam after a median treatment duration of 38 months (range, 23-69 months), 4 (80%) due to bulbar deterioration or lack of motor improvement and 1 (20%) due to injection difficulties.

### Respiratory Function and Nutritional Support

**Figure 1.**
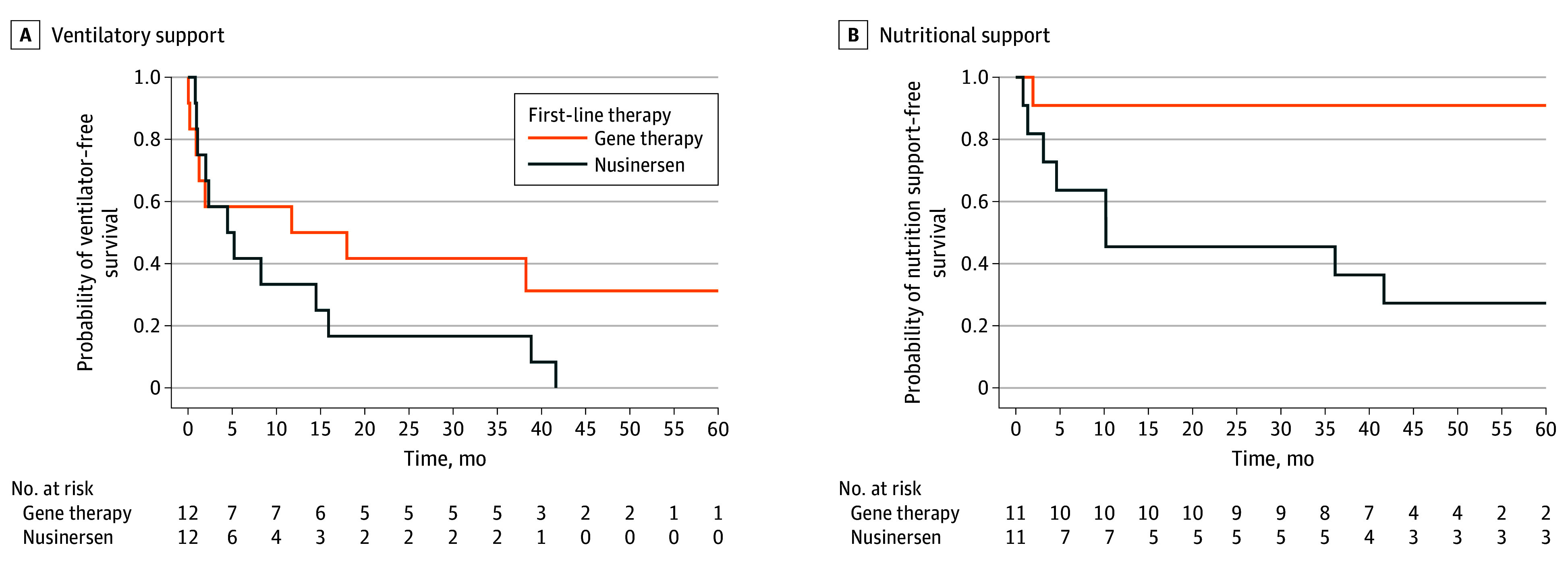
Kaplan-Meier Curves Showing Time to Initiation of Ventilatory and Nutritional Support After Treatment Initiation The graphs show time in months between treatment initiation and support initiation for patients alive at the cutoff date or between treatment initiation and death for patients who died by the cutoff date. At each time point, the number at risk included patients alive without ventilation or nutrition support; patients who had not reached the time point were censored. B, Two patients who required feeding support shortly before initiating treatment (13 days before nusinersen and 8 days before gene therapy) were excluded from the analysis.

In both groups, most ventilatory support needs arose within the first year of treatment (Figure 1A and eTable 3 in Supplement 1). At 2 years posttreatment, 5 of the 11 surviving patients in the gene therapy group (45%) and 8 of the 10 surviving patients in the nusinersen group (80%) required ventilatory support, all limited to sleep. Two of those children in the gene therapy group (40%) were weaned off nocturnal ventilation; none in the nusinersen group improved.

At baseline, 11 patients in each group did not require nutritional support. During follow-up, none of those 11 patients in the gene therapy group required new nutritional support. In contrast, in the nusinersen group, 5 of the 11 patients (45%) who did not require support at baseline subsequently needed nutritional support after treatment initiation. At 2 years posttreatment, 1 of the 11 surviving patients treated with gene therapy (9%) required nutritional support vs 5 of 10 (50%) treated with nusinersen. Two of the 10 surviving patients in the nusinersen group (20%) developed severe bulbar dysfunction, rendering them unable to eat orally or swallow saliva. Within-pair growth trajectories (eFigure 2 in Supplement 1) were similar except for pairs 1 and 12, probably owing to nutritional support needs.

### Motor Outcomes

**Figure 2.**
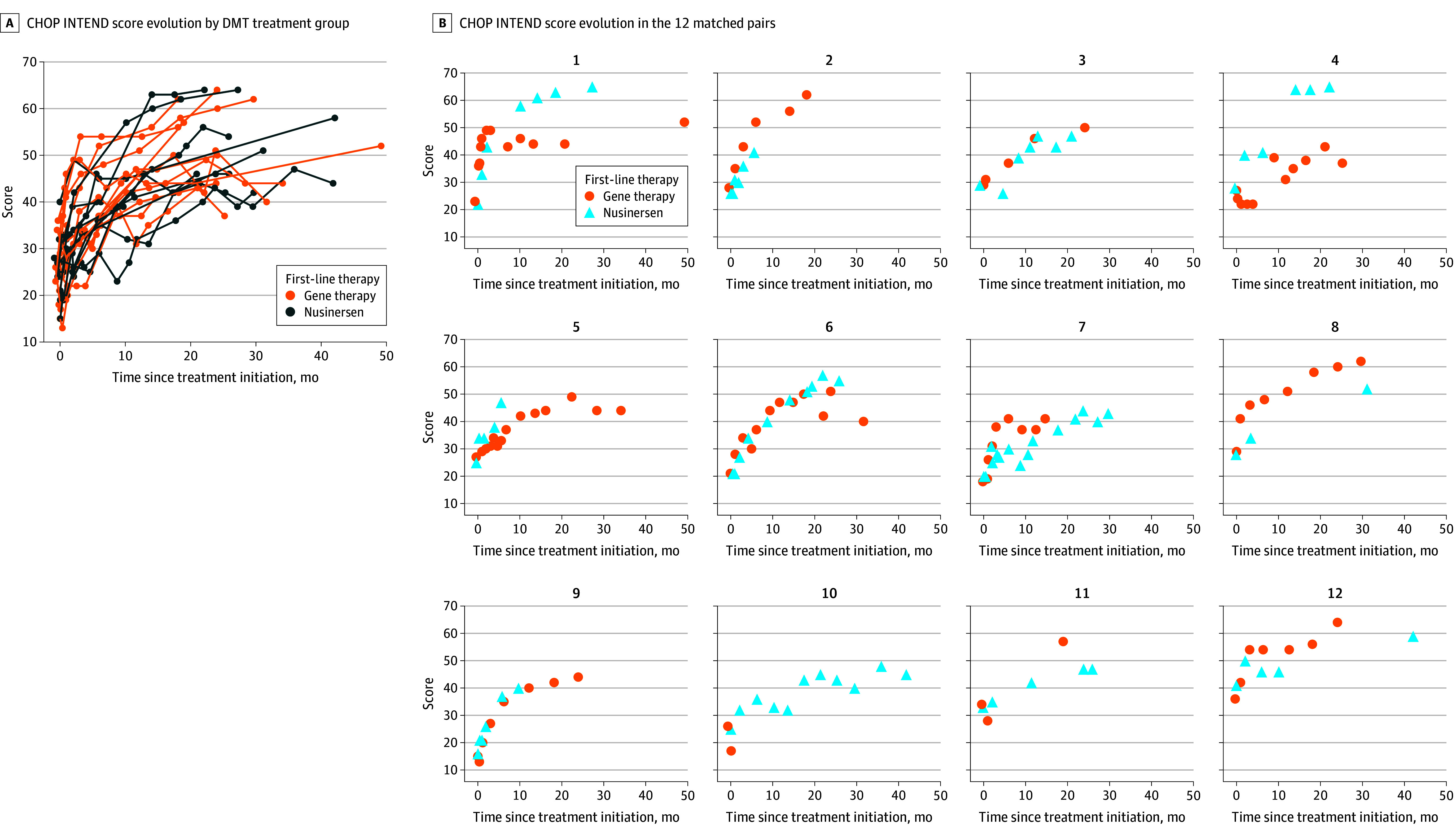
Children’s Hospital of Philadelphia Infant Test of Neuromuscular Disorders (CHOP INTEND) Score Evolution Over Time Since Initiation and by Matched Pairs The CHOP INTEND score ranges from 0 to 64, with higher scores indicating better function. B, The patients treated with nusinersen in pairs 2 and 9 and the patient treated with gene therapy in pair 10 died during the first year of treatment, explaining the truncated follow-up. DMT indicates disease-modifying treatment.

No motor skill regression occurred. The 2 linear mixed models used to analyze time as either months since treatment initiation or log (1 + months since treatment initiation) yielded similar results. To facilitate interpretation, only the curves and results using time defined as months since treatment initiation are presented (Figure 2). All surviving patients in both cohorts achieved a minimal clinically important difference of 4 or more points in CHOP INTEND score from baseline. No significant differences were observed in mean (SE) CHOP INTEND score change from the index date between the gene therapy and nusinersen groups. The estimated monthly increase in CHOP-INTEND scores was 0.82 points (95% CI, 0.56-1.1 points) in the gene therapy group and 1.15 points (95% CI, 0.71-1.65 points) in the nusinersen group. In the paired analysis, no significant difference was observed in the mean change from baseline between patients treated with nusinersen and those treated with gene therapy (mean [SE] paired difference, −1.69 [1.24] points; *P* = .17).

**Figure 3.**
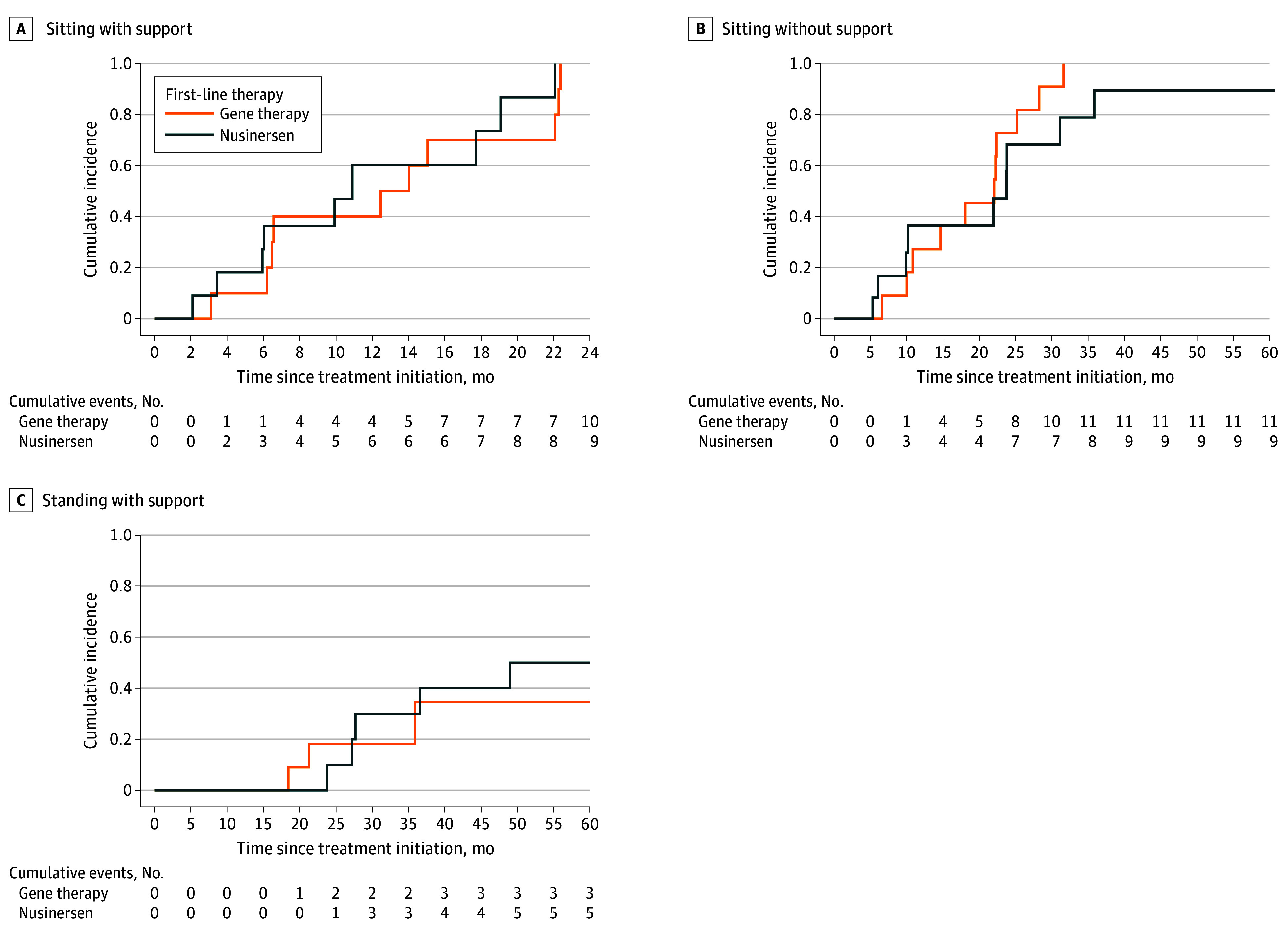
Kaplan-Meier Curves Showing Time to Achievement of Supported Sitting and Standing and Independent Sitting No statistical comparison between groups was performed, and CIs were omitted due to the limited number of events and small sample size. At each time point, the number of events reflects the number of surviving patients who achieved the analyzed motor ability. A, One patient with spinal muscular atrophy type 1c (SMA1c) in the nusinersen group and 1 with SMA1c in the gene therapy group achieved this milestone 42 days and 36 days, respectively, before treatment initiation and were excluded from the Kaplan-Meier analysis to avoid potential immortal time bias and ensure comparability across treatment groups. B, One patient in the nusinersen group did not achieve the milestone; this patient lacked motor capacity at baseline, gained the ability to sit with support 18 months after nusinersen initiation, and showed no further motor progress during 5 years of follow-up.

Motor developmental milestones in the matched pairs are summarized in Figure 3 and eTable 4 in Supplement 1. Kaplan-Meier curves showed similar patterns of motor milestone achievements. At 2 years after DMT initiation, all surviving patients could sit with support. Independent sitting was achieved by all patients treated with gene therapy and 9 of 10 patients treated with nusinersen (90%).

### Unsatisfactory Clinical Response

The UCR rate was higher in the nusinersen (8 of 12 patients [67%]) than in the gene therapy (3 of 12 patients [25%]) group, with a crude matched odds ratio (OR) of 2.67 (95% CI, 0.71-10.05) (exact McNemar test OR, 6.00; 95% CI, 0.73-275.64). These results were consistent with those of the conditional logistic regression model. UCR events included death (1 patient [8%] in each group), need for nutritional support (7 [58%] in the nusinersen group), and add-on therapy (2 [17%] in the gene therapy group) (eTable 5 in Supplement 1).

## Discussion

To our knowledge, this study provides the first comparative analysis of nusinersen and gene therapy as first-line DMTs in children with SMA1, using rigorously matched cohorts from the French National SMA Registry. Despite the small sample size, our study offers valuable insights into the comparative outcomes of these therapies in routine clinical practice, highlighting both their transformative potential and important distinctions in clinical outcomes, particularly regarding UCR and respiratory and nutritional outcomes. Motor improvements were largely comparable, suggesting both options may be valid DMTs, though differences emerged in other clinically relevant domains. All surviving patients in both cohorts achieved a minimal clinically important difference of 4 or more points in CHOP INTEND score and reached the ability to sit independently. These findings align with prior registry studies and suggest the motor efficacy of both drugs in symptomatic infants, although comparisons are hampered by population heterogeneity across studies.^26,27^

Our design aimed to address the methodologic challenges of clinical studies by applying rigorous 1:1 matching on clinically meaningful baseline variables (ie, age, CHOP INTEND score, and ventilatory and feeding status) and by relying on exact methods for binary outcomes.^28^ Although not statistically significant, differences in ventilatory and feeding outcomes were found. Patients treated with nusinersen had higher rates of ventilatory and nutritional support than those treated with gene therapy, as well as more frequent UCR, despite equivalent baseline symptom severity. One plausible explanation may be the lower bioavailability of nusinersen in brainstem motor nuclei, as previously hypothesized.^29^ These observations are consistent with prior studies suggesting potentially superior bulbar and respiratory outcomes of gene therapy compared with nusinersen.^9,11^ Although exploratory and based on small numbers, such differences are relevant in guiding early treatment decisions and anticipating long-term needs in SMA1 care.

The more frequent UCR in the nusinersen group highlights the challenges of achieving sustained efficacy in some patients. In practice, these reevaluations often reflect suboptimal disease control and precede therapeutic escalation. While both therapies yielded similar motor improvements, our results highlight persistent challenges in addressing bulbar and respiratory dysfunction, particularly in symptomatic infants. Our findings also reinforce the importance of early treatment initiation, as both therapies are more effective when administered before symptom onset.^9,13,30,31,32^

The mortality rate in this study (3 of 24 patients [13%]) was higher than that reported in the literature for children with SMA1 treated with nusinersen (5%) or gene therapy (1.7%).^9,11,26^ These discrepancies may reflect more severe disease at treatment initiation and may also relate to longer follow-up or differences in cohort composition compared with the prior reports. All deaths in this study were attributed to disease severity rather than treatment failure.

Several questions remain regarding the optimal combination or sequencing of therapies. Direct assessment of neuronal transfection rates in gene therapy is not feasible in clinical practice, representing a key limitation in evaluating its full therapeutic potential. Conversely, optimizing nusinersen delivery or dosing to improve bulbar and respiratory efficacy also warrants further investigation.^33,34^ Additionally, the differential impact of both therapies on expressive speech, swallowing, and respiratory function should be explored in future studies. As SMA newborn screening programs are being progressively implemented, head-to-head studies including presymptomatic infants will be essential to guide individualized treatment decisions within narrow therapeutic windows. Our findings, although exploratory, support gene therapy as a promising first-line option in symptomatic SMA1, particularly for children at risk of early bulbar or respiratory involvement.

### Limitations

This study has several limitations inherent to registry-based observational data.^27^ First, despite rigorous 1:1 matching on clinically meaningful baseline variables (age, motor function, and respiratory and nutritional status) to ensure comparability between treatment groups and reduce confounding by indication, residual confounding cannot be excluded.^21^ Propensity score methods, although widely used in larger studies, were avoided herein due to limited sample size and the risk of imbalanced or clinically dissimilar matches.^20,21,22,35,36^ Instead, exact matching on pretreatment characteristics was preferred for interpretability and internal validity. Second, treatment initiation occurred in different calendar periods. Although SMA care standards have remained stable in France since 2017, residual era effects are possible. Potential era effects were mitigated through (1) matching on baseline severity and (2) the stability of national SMA care guidelines since 2017 regarding respiratory, nutritional, and motor management. Third, statistical power was limited. We used exact statistical methods for binary outcomes (eg, McNemar test) rather than covariate-adjusted models, which would have been underpowered and potentially unstable due to the limited number of matched pairs (n = 12). Fourth, outcome measures such as respiratory and nutritional support were pragmatic and clinically meaningful but may lack the granularity of standardized scales. However, the French National SMA Registry provides high-quality, longitudinal data collected through standardized procedures, enhancing reliability and minimizing misclassification.^18^ Fifth, our cohort included only symptomatic infants. The applicability of these findings to presymptomatic patients or other phenotypes (eg, SMA type 2) remains limited. Future studies targeting presymptomatic infants treated within a narrow therapeutic window are needed to refine treatment recommendations in newborn screening programs.

## Conclusions

Although based on a small number of matched pairs, the findings of this comparative effectiveness study suggest that gene therapy may be associated with lower incidence of UCR and less need for supportive care compared with nusinersen in symptomatic children with SMA type 1, while motor function outcomes appeared similar. These exploratory findings, while not statistically conclusive, support further investigation and suggest the consideration of gene therapy as a preferred first-line option in this population. The findings may help inform first-line treatment choices in this rare and severe disease. For other SMA phenotypes, the decision between gene therapy and nusinersen should remain a shared process that carefully balances the benefits and potential burdens of each approved therapy.

## Notes

Supplement 1. **eTable 1.** Pairwise Matching Characteristics for 1:1 Matching of SMA1 Patients Treated With Gene Therapy or Nusinersen **eMethods.** Statistical Analysis Plan **eFigure 1.** Flowchart **eTable 2.** Characteristics of the Unmatched Patients at Index Date According to the First-Line Therapy **eFigure 2.** Weight Over Time Since Treatment Initiation **eTable 3.** Individual-Level Data Used to Generate Kaplan-Meier Ventilation-Free and Nutrition Support–Free Survival Curves **eTable 4.** Maximal Motor Abilities Over Time in Matched Patients According to the First-Line Therapy **eTable 5.** Unsatisfactory Clinical Response

Supplement 2.
Members of the French SMA Registry Study Group

Supplement 3.
Data Sharing Statement
