# Supplementary material for: Comparative Clinical Outcomes of Nusinersen and Gene Therapy in Spinal Muscular Atrophy Type 1
Source: JAMA Netw Open. 2025 Oct 8;8(10):e2536348. doi: 10.1001/jamanetworkopen.2025.36348 (PMC12508997; doi:10.1001/jamanetworkopen.2025.36348)
Supplement: Supplement 1. — eTable 1. Pairwise Matching Characteristics for 1:1 Matching of SMA1 Patients Treated With Gene Therapy or Nusinersen eMethods. Statistical Analysis Plan eFigure 1. Flowchart eTable 2. Characteristics of the Unmatched Patients at Index Date According to the First-Line Therapy eFigure 2. Weight Over Time Since Treatment Initiation eTable 3. Individual-Level Data Used to Generate Kaplan-Meier Ventilation-Free and Nutrition Support–Free Survival Curves eTable 4. Maximal Motor Abilities Over Time in Matched Patients According to the First-Line Therapy eTable 5. Unsatisfactory Clinical Response [file jamanetwopen-e2536348-s001.pdf]

# Supplementary Online Content

Ropars J, Cances C, Garcia-Uzquiano R, et al; for the French SMA Registry Study Group. Comparative clinical outcomes of nusinersen and gene therapy in spinal muscular atrophy type 1. *JAMA Netw Open*. 2025;8(10):e2536348. doi:10.1001/jamanetworkopen.2025.36348

**eTable 1.** Pairwise Matching Characteristics for 1:1 Matching of SMA1 Patients Treated With Gene Therapy or Nusinersen

**eMethods.** Statistical Analysis Plan

**eFigure 1.** Flowchart

**eTable 2.** Characteristics of the Unmatched Patients at Index Date According to the First-Line Therapy

**eFigure 2.** Weight Over Time Since Treatment Initiation

**eTable 3.** Individual-Level Data Used to Generate Kaplan-Meier Ventilation-Free and Nutrition Support–Free Survival Curves

**eTable 4.** Maximal Motor Abilities Over Time in Matched Patients According to the First-Line Therapy

**eTable 5.** Unsatisfactory Clinical Response

This supplementary material has been provided by the authors to give readers additional information about their work.

© 2025 Ropars J et al. *JAMA Network Open*.

# eTable 1: Pairwise matching characteristics for 1:1 matching of SMA1 patients treated with gene therapy or nusinersen

| Pair ID | Treatment Group | Age at treatment (days) | CHOP INTEND score | Ventilatory support (Yes/No) | Feeding support (Yes/No) |
|---|---|---|---|---|---|
| 1 | NsN | 85.00 | 21 | No | No |
| 1 | GTx | 70.04 | 23 | No | No |
| 2 | GTx | 127.04 | 28 | No | No |
| 2 | NsN | 130.96 | 25 | No | No |
| 3 | GTx | 132.96 | 29 | No | No |
| 3 | NsN | 112.96 | 28 | No | No |
| 4 | GTx | 130.04 | 27 | No | Yes |
| 4 | NsN | 121.04 | 27 | No | Yes |
| 5 | GTx | 281.00 | 27 | No | No |
| 5 | NsN | 266.00 | 24 | No | No |
| 6 | GTx | 161.00 | 21 | No | No |
| 6 | NsN | 177.04 | 20 | No | No |
| 7 | GTx | 147.04 | 18 | No | No |
| 7 | NsN | 153.96 | 19 | No | No |
| 8 | GTx | 307.00 | 29 | No | No |
| 8 | NsN | 327.00 | 27 | No | No |
| 9 | GTx | 117.96 | 15 | No | No |
| 9 | NsN | 132.96 | 15 | No | No |
| 10 | NsN | 98.04 | 24 | No | No |
| 10 | GTx | 127.04 | 26 | No | No |
| 11 | NsN | 338.00 | 32 | No | No |
| 11 | GTx | 360.00 | 34 | No | No |
| 12 | GTx | 258.04 | 36 | No | No |
| 12 | NsN | 274.00 | 40 | No | No |

*Legend: Age at treatment, CHOP INTEND score, and ventilatory/feeding support were recorded at treatment initiation and used for 1:1 exact matching within predefined tolerance intervals (see Methods).*

© 2025 Ropars J et al. *JAMA Network Open*.

# eMethods

# Comparative Effectiveness of Nusinersen and Gene therapy in children with spinal muscular atrophy type 1: A matched cohort study using real-world data from the French SMA Registry

## Statistical Analysis Plan


| | |
|---|---|
| Principal investigator | Juliette Ropars, MD, PhD<br>Centre Neuromusculaire<br>Service de Pédiatrie<br>CHU Brest<br>Brest, France<br><br>LaTIM<br>INSERM UMR 1101<br>Brest, France<br><br>Unité de Recherche Clinique APHP<br>Université Paris-Saclay<br>Direction de la Recherche Clinique<br>Assistance Publique Hôpitaux de Paris<br>Paris, France |
| Authors | Lionelle Nkam and Lamiae Grimaldi<br>Statistician and methodologist<br>Clinical Research Unit, Ambroise Paré Hospital,<br>Boulogne-Billancourt, France |


| | |
|---|---|
| Version | 1.0 |
| Version date | 01/10/2022 |

© 2025 Ropars J et al. *JAMA Network Open*.

## Table of contents


1. Introduction ........ 7
2. Study context ........ 7
3. Study objectives ........ 7
4. Study design ........ 7
5. Data source and study population ........ 7
   - 5.1. Inclusion criteria ........ 7
   - 5.2. Exclusion criteria ........ 8
6. Statistical methods ........ 8
   - 6.1. Index date ........ 8
   - 6.2. Matching criteria ........ 8
   - 6.3. Descriptive analysis ........ 8
   - 6.4. Baseline assessment and last assessment ........ 9
   - 6.5. Follow-up assessment measures ........ 9
   - 6.6. Motor abilities ........ 9
   - 6.7. Ventilatory status ........ 10
   - 6.8. Feeding status: ........ 10
   - 6.9. Unsatisfactory clinical response analysis ........ 10
   - 6.10. Motor function analysis ........ 10
7. Flowchart and tables ........ 11
   - 7.1. Flowchart ........ 11
   - 7.2. Tables ........ 12


© 2025 Ropars J et al. *JAMA Network Open*.

# List of figures

Figure 1: Selection of patients in the study ........ 11

# List of tables

Table 1 : Characteristics of the matched patients at index date according to the first line therapy.... 12
Table 2 : Follow-up time, treatment changes and vital status in each treated population ........ 13
Table 3 : Association between treatment failure and first line therapy ........ 14

© 2025 Ropars J et al. *JAMA Network Open*.

# List of of abbreviations

DMT: disease-modifying therapy

GTx: gene therapy (onasemnogene abeparvovec)

NsN: Nusinersen 14 mg

SMA: Spinal Muscular Atrophy

SAP: Statistical Analysis Plan

© 2025 Ropars J et al. *JAMA Network Open*.

# 1. INTRODUCTION

This statistical analysis plan (SAP) outlines the planned analyses for the study. The results presented in the final clinical study report will follow the strategy described in this document. Any deviations from this SAP will be documented and justified in the final report. Any additional analyses will be detailed in a separate analysis plan.

The analysis will be conducted by the Clinical Research Unit Paris Saclay Ouest under the supervision of Pr Lamiae Grimaldi. It will be carried out by a qualified and experienced statistician (Lionelle NKAM) and methodologist (Lamiae Grimaldi) to ensure the integrity of the data.

# 2. STUDY CONTEXT

Therapeutic advances have transformed the prognosis of Spinal Muscular Atrophy (SMA), significantly increasing life expectancy and altering the natural history of this neurodegenerative disease. Given the lifelong impact of these innovative therapies, comparative data on their real-world effectiveness are essential.

# 3. STUDY OBJECTIVES

The objective of this study is to compare the effectiveness of nusinersen (NsN) and onasemnogene abeparvovec gene therapy (GTx) in children with SMA type 1.

# 4. STUDY DESIGN

This study is designed as a matched cohort study comparing children with SMA type 1 who are exposed to GTx versus those exposed to NsN.

# 5. DATA SOURCE AND STUDY POPULATION

The study population will consist of all patients included in the French SMA registry up to July 22, 2024 (Date of data extractionand who received either GTx or NsN as a first line disease-modifying therapy (DMT).

The French SMA registry is an observational, national, multi-centre disease registry that includes patients with genetically confirmed 5q SMA. This registry collects longitudinal data – both historically and prospectively – in real-life settings on all available SMA patients, regardless of their treatment regimen.

## 5.1. Inclusion criteria

Patients meeting the following criteria at treatment initiation will be included in the study:

- SMA type 1
    - SMA type 1 A and SMA type 1 B aged less than 9 months at treatment initiation
    - SMA type 1 C aged less than 12 months at treatment initiation
- Presence of 2 or 3 copies of SMN2

© 2025 Ropars J et al. *JAMA Network Open*.

- Incident users of GTx or NsN, naïve to prior DMT (patients who received a bridging treatment before GTx will be excluded). (patients who received a bridging treatment before GTx will be excluded)
- Treatment initiation within 6 months after genetic diagnosis
- At least 24 months of follow-up post-treatment initiation, on July 22, 2024

### 5.2. Exclusion criteria

Patients will be excluded if they:

- Were diagnosed pre-symptomatically.
- Have less than 2 copies or more than 3 copies of SMN2.Have SMA type 2, type 3 or type 4
- Received their treatment as part of the the SMART, STRENGHT or STRIVE randomised clinical trials

## 6. STATISTICAL METHODS

Statistical analyses will be performed after the database is frozen (on July 22, 2024). Data will be analyzed using R software (R version 4.1.1 (2021-08-10), The R Foundation for Statistical Computing).

### 6.1. Index date

The index date is defined as the date of initiation of the first-line DMT (GTx or NsN).

### 6.2. Matching criteria

Patients who received GTx as a first-line DMT will be matched to those who received NsN as a first-line DMT on the following criteria:

- Age at treatment initiation (matching window):
  - If age at treatment initiation is ≤ 30 days: ±7 days
  - If age at treatment initiation is > 30 days and ≤ 90 days: ±15 days
  - If age at treatment initiation is > 90 days : ±30days
- CHOP INTEND at treatment initiation (matching window):
  - If CHOP INTEND at treatment initiation is < 30 : ±3 points
  - If CHOP INTEND at treatment initiation is >= 30 : ±5 points
- Feeding status at treatment initiation: Yes / No
- Ventilatory status at treatment initiation: Yes / No

### 6.3. Descriptive analysis

Continuous data will be summarized using themean, standard deviation, minimum, 25th percentile (Q1), median, 75th percentile (Q3), and maximum, along withthe number of complete and missing observations. Categorical data will be summarized by the total number of patients in each category and the number of missing values. Relative frequencies will be presented as overall percentages.

© 2025 Ropars J et al. *JAMA Network Open*.

### 6.4. Baseline assessment and last assessment

Baseline assessment will be defined as the measurements recorded at the index date. If no assessment is available at the index date, the closest assessment within 45 days prior to or 15 days after the index date will be considered, with preference given to assessments prior to the index date.

An exception will be made for ventilation status, where no time window prior to the index date will be considered. If no ventilation assessment is available at the index date, the last available assessment before the index date or the first assessment within 15 days after will be used as the baseline, with preference given to assessments before the index date.

If no assessment is found within these time intervals, the value of the variable was considered as missing in the description.

### 6.5. Follow-up assessment measures

Follow-up assessments are assessments measured at predefined time points during the study period, specifically:

- 12 months after the index date ± 1 month
- 24 months after the index date ± 2 months
- 36 months after the index date ±3 months
- 48 months after the index date ±3 months
- 60 months after the index date ±3 months
- 72 months after the index date ±3 months
- 84 months after the index date ±3 months

The closest available assessment to each of these time points will be used. If no assessment is available at the specific time, the most recent assessment prior to that time considered will be considered.

### 6.6. Motor abilities

Motor abilities are ranked in order of progression, from ‘’no capacity acquired’’ to ‘’running’’ as follows:

1) No capacity acquired
2) Head control (more than 3 seconds)
3) Rolling to one side
4) Rolling completely
5) Sitting with support
6) Sitting without support (30 seconds)
7) Crawling
8) Walking on four limbs
9) Standing with support
10) Standing without support (60 seconds)
11) Walking with support
12) Walking without support (10 meters)
13) Climbing stairs
14) Running

© 2025 Ropars J et al. *JAMA Network Open*.

For example, if a patient had no acquired motor skills at baseline but was later reported as sitting without support, this implies that this patient had also achieved sitting with support, rolling on both sides and head control.

### 6.7. Ventilatory status

Ventilatory status will be described as follows:

- No ventilatory support of any kind
- Invasive ventilation via tracheotomy
  - during sleep only
  - more than 16 hours per day
  - less than 3 hours per day
- Non-invasive ventilation
  - during sleep only
  - more than 16 hours per day
  - less than 3 hours per day

Note that only ventilation lasting at least 31 consecutive days will be considered.

### 6.8. Feeding status:

Feeding status will be categorized as follows:

- Oral feeding only
- Nasogastric tube
- Gastrostomy button
- Gastrojejunal tube
- Other type of feeding

Note: Only enteral feeds lasting at least 31 consecutive days will be considered.

### 6.9. Unsatisfactory clinical response analysis

Unsatisfactory clinical response is defined as death, switching to another therapy due to inadequate response, initiation of nutritional support, or failure to achieve of the ability to sit unaided.

The unsatisfactory clinical response will be analyzed within a maximum follow-up period of 60 months after treatment initiation. The failure event will be observed within the same time window for both patients in a matching pair corresponding to the shortest follow-up time between them, except when the failure event is death. If one patient in the matching pair dies, the other patient will be followed until unsatisfactory clinical response or end of follow-up, whichever comes first.

A conditional logistic regression model stratified by matching pairs, will be used to analyze unsatisfactory clinical response. The main independent variable will be first-line DMT, with year of treatment included as an adjustment variable.

### 6.10. Motor function analysis

A linear mixed model will be used to assess the evolution of CHOP-INTEND score changes from baseline over time, according to first line DMT. This model will account for nested random

slopes considering both the subject identifier and the matched pair. In addition, two different specifications of the time variable will be tested : time = months since treatment initiation; and time = log (1 + months since treatment initiation).

# 7. FLOWCHART AND TABLES

## 7.1. Flowchart

A flowchart detailing patient selection will be provided as follows.

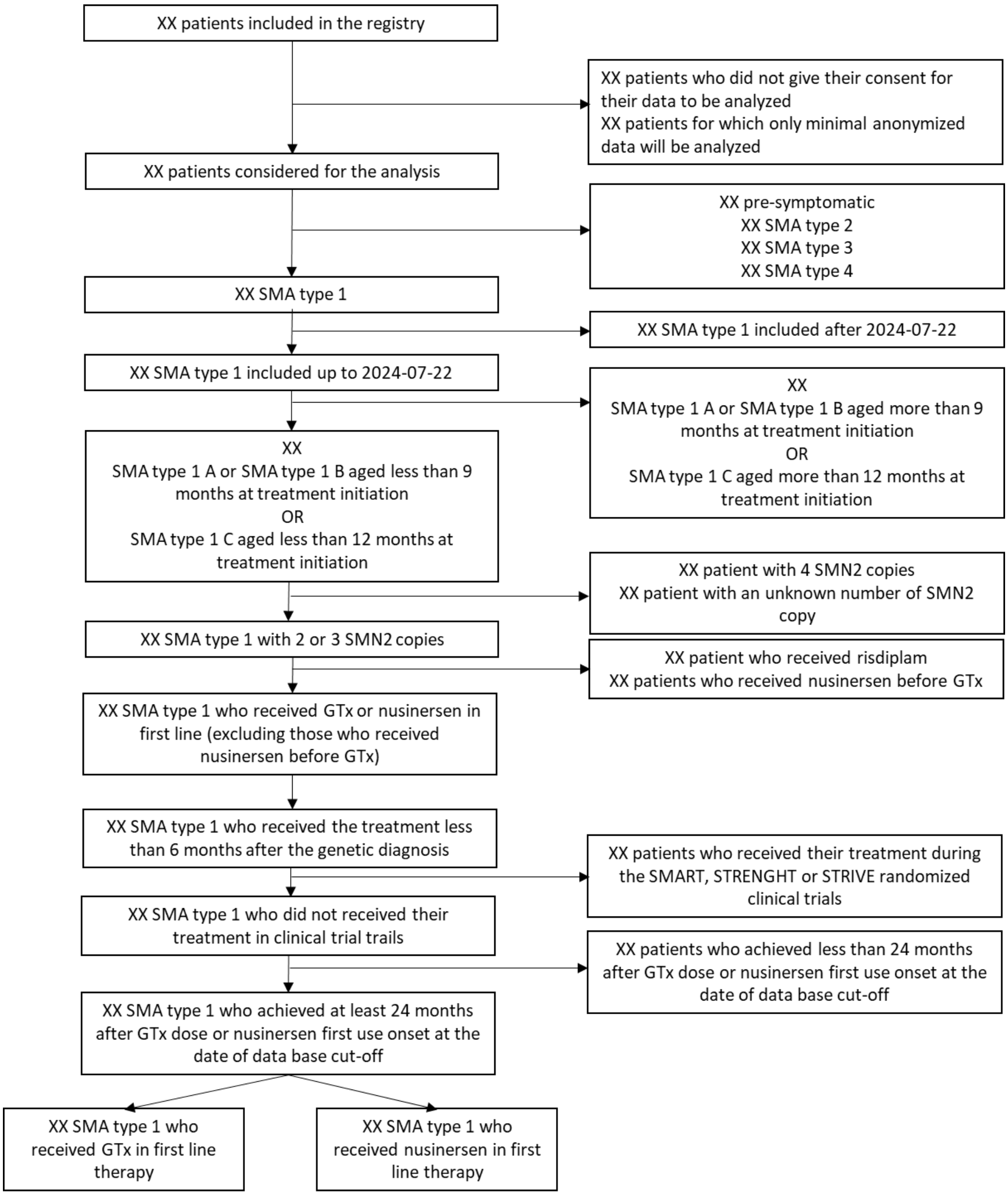


**Figure 1: Selection of patients in the study**

© 2025 Ropars J et al. *JAMA Network Open*.

## 7.2. Tables

**Table 1 : Characteristics of the matched patients at index date according to the first line therapy**

| Variables | Matched Gene Therapy population N= | Matched nusinersen population N= |
|---|---|---|
| Gender | | |
| Male (%) | | |
| Female (%) | | |
| Age at first treatment initiation (months)* | | |
| mean(std) | | |
| med[IQ] | | |
| min-max | | |
| Type of SMA | | |
| SMA 1A (%) | | |
| SMA 1B (%) | | |
| SMA 1C (%) | | |
| Number of SMN2 copies | | |
| 2 copies (N, %) | | |
| 3 copies (N, %) | | |
| Age at symptom onset (months) | | |
| mean(std) | | |
| med[IQ] | | |
| min-max | | |
| Age at genetic diagnosis (months) | | |
| mean(std) | | |
| med[IQ] | | |
| min-max | | |
| Weight at first treatment initiation (kg) | | |
| mean(std) | | |
| med[IQ] | | |
| min-max | | |
| Ventilatory support of any type at baseline* | | |
| No (%) | | |
| Feeding support at baseline* | | |
| Oral feeding only (%) | | |
| Feeding support (%) | | |
| CHOP-INTEND score at treatment initiation (1)* | | |
| mean(std) | | |
| med[IQ] | | |
| min-max | | |
| CHOP-INTEND score at treatment initiation (2) | | |
| CHOP >= 15 and CHOP < 30 (%) | | |
| CHOP >= 30 and CHOP < 45 (%) | | |
| Maximal motor ability at treatment initiation | | |
| No capacity (%) | | |
| Head control (more than 3sec) (%) | | |
| Sitting with support (%) | | |
| Acquisition age at maximal motor ability before treatment initiation (months) | | |
| mean(std) | | |
| med[IQ] | | |
| min-max | | |

*Matching criteria

© 2025 Ropars J et al. *JAMA Network Open*.

Table 2 : Follow-up time, treatment changes and vital status in each treated population

| Variables | Matched Gene Therapy population N= | Matched nusinersen population N= |
| --- | --- | --- |
| Follow-up time from first treatment initiation to last assessment or death (months) :<br>Defined as the difference between date of last assessment and date of first treatment for patients alive at data base cut-off<br>Defined as the dfference between date of death and date of first treatment for patients deceased at data base cut-off<br>mean(std)<br>med[IQ]<br>min-max | | |
| Follow-up time from first treatment initiation to last assessment or death (months) in classes<br>Follow-up < 12 months (%)<br>Follow-up >= 24 months and < 36 months (%)<br>Follow-up >= 36 months and < 48 months (%)<br>Follow-up >= 48 months and < 60 months (%)<br>Follow-up >= 60 months (%) | | |
| GTx bridge towards nusinersen<br>Yes (N, %) | | |
| nusinersen switch to risdiplam or GTx bridge to risdiplam<br>Yes (N, %) | | |
| Vital status at data base cut-off<br>Deceased (N, %) | | |
| Age at death (years)<br>mean(std)<br>med[IQ]<br>min-max | | |

© 2025 Ropars J et al. *JAMA Network Open*.

Table 3 : Association between unsatisfactory clinical response and first line therapy

| **First line treatment** | Unsatisfactory clinical response | Crude matched Odds Ratio [95% CI][a] |
|---|---|---|
| GTx N(%) | | |
| Nusinersen N(%) | | |

[a]: Conditional logistic regression model

CI: confidence interval

# eFigure 1. Flowchart

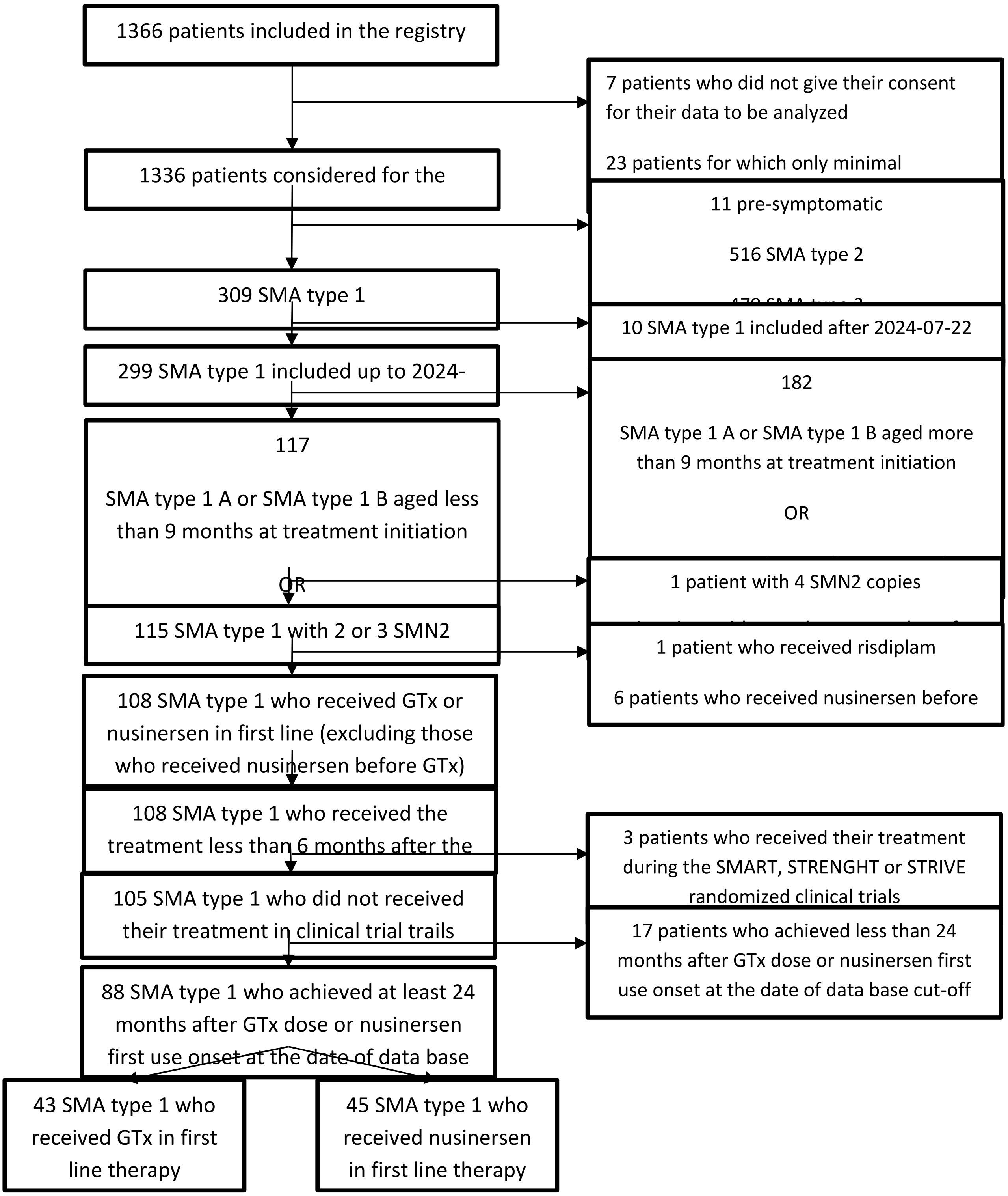


© 2025 Ropars J et al. *JAMA Network Open*.

## eTable 2: Characteristics of the unmatched patients at index date according to the first-line therapy

| Variables | Statistics | Unmatched Gene Therapy population | Unmatched nusinersen population |
|---|---|---|---|
| Gender | N | 31 | 33 |
| | Male (%) | 11 (35.5) | 17 (51.5) |
| | Female (%) | 20 (64.5) | 16 (48.5) |
| Age at first treatment initiation (months) : Defined as the difference between date at first treatment initiation and date of birth | N | 31 | 33 |
| | mean(std) | 5.8(3.2) | 5.8(3) |
| | med[IQ] | 5.6[2.9;8.2] | 5.4[3.7;7.9] |
| | min-max | 0.9-11.5 | 1.7-11.9 |
| Type of SMA (1) | N | 31 | 33 |
| | SMA 1A (%) | 2 ( 6.5) | 6 (18.2) |
| | SMA 1B (%) | 18 (54.5) | 16 (51.6) |
| | SMA 1C (%) | 11 (35.5) | 11 (33.3) |
| Type of SMA (2) | N | 31 | 33 |
| | SMA 1C (%) | 11 (35.5) | 11 (33.3) |
| Number of SMN2 copies | N | 31 | 33 |
| | 2 Copies (%) | 26 (83.9) | 28 (84.8) |
| | 3 Copies (%) | 5 (16.1) | 5 (15.2) |
| Age at symptom onset (months) | N | 31 | 31 |
| | mean(std) | 2.9(2.1) | 2.6(2.1) |
| | med[IQ] | 3.0[1.4;4.0] | 1.6[1.0;4.3] |
| | min-max | 0.0-9.1 | 0.0-7.0 |
| | Missing | 0 | 2 |
| Age at genetic diagnosis (months) | N | 31 | 33 |
| | mean(std) | 5.2(3) | 4.8(2.8) |
| | med[IQ] | 4.9[2.4;7.5] | 4.0[2.2;7.0] |
| | min-max | 0.4-11.0 | 1.5-10.9 |
| Weight at first treatment initiation (kg) | N | 31 | 31 |
| | mean(std) | 6.7(1.4) | 6.4(1.7) |
| | med[IQ] | 6.4[5.6;7.5] | 6.4[5.4;7.7] |
| | min-max | 4.2- 9.7 | 3.8-11.0 |
| | Missing | 0 | 2 |
| Ventilatory support of any type at baseline | N | 31 | 33 |
| | No (%) | 26 (83.9) | 20 (60.6) |
| | Yes (%) | 5 (16.1) | 13 (39.4) |
| Type of ventilatory support at baseline | N | 5 | 13 |
| | Non invasive ventilation (%) | 5 (100.0) | 13 (100.0) |
| Frequence of ventilatory support at baseline | N | 5 | 13 |
| | Noninvasive ventilation during sleep only (%) | 5 (100.0) | 10 ( 76.9) |
| | Noninvasive ventilation | 0 ( 0.0) | 3 (23.1) |

© 2025 Ropars J et al. *JAMA Network Open*.

| Variables | Statistics | Unmatched Gene Therapy population | Unmatched nusinersen population |
|---|---|---|---|
| | more than 16 hours per day (%) | | |
| Feeding support at baseline | N | 31 | 33 |
| | Oral feeding only (%) | 29 (93.5) | 18 (54.5) |
| | Nasogastric tube (%) | 2 ( 6.5) | 13 (39.4) |
| | Gastrostomy button (%) | 0 (0.0) | 2 (6.1) |
| CHOP-INTEND score at treatment initiation (1) | N | 28 | 16 |
| | mean(std) | 25.4(10) | 26.8(12.7) |
| | med[IQ] | 25.5[17.8;32.5] | 26.0[19.5;31.2] |
| | min-max | 9.0-50.0 | 8.0-53.0 |
| | Missing | 3 | 17 |
| CHOP-INTEND score at treatment initiation (2) | N | 28 | 16 |
| | CHOP < 15 (%) | 4 (12.9) | 2 ( 6.1) |
| | CHOP >= 15 and CHOP < 30 (%) | 15 (48.4) | 7 (21.2) |
| | CHOP >= 30 and CHOP < 45 (%) | 8 (25.8) | 5 (15.2) |
| | CHOP >= 45 (%) | 1 (3.2) | 2 (6.1) |
| | Missing (%) | 3 ( 9.7) | 17 (51.5) |
| Maximal milestone at treatment initiation | N | 31 | 31 |
| | No capacity (%) | 16 (51.6) | 22 (66.7) |
| | Head control (more than 3 sec) (%) | 11 (35.5) | 7 (21.2) |
| | Sitting with support (%) | 3 (9.7) | 2 (6.1) |
| | Sitting without support (%) | 1 (3.2) | 0 (0.0) |
| | Missing (%) | 0 (0.0) | 2 (6.1) |
| Age at Maximal milestone at treatment initiation (months) | N | 31 | 31 |
| | mean(std) | 5.7(3.2) | 5.7(2.9) |
| | med[IQ] | 5.4[2.8;8.2] | 4.7[4.0;7.6] |
| | min-max | 0.9-11.3 | 1.7-11.9 |
| | Missing | 0 | 2 |

© 2025 Ropars J et al. *JAMA Network Open*.

# eFigure 2: Weight over time since treatment initiation

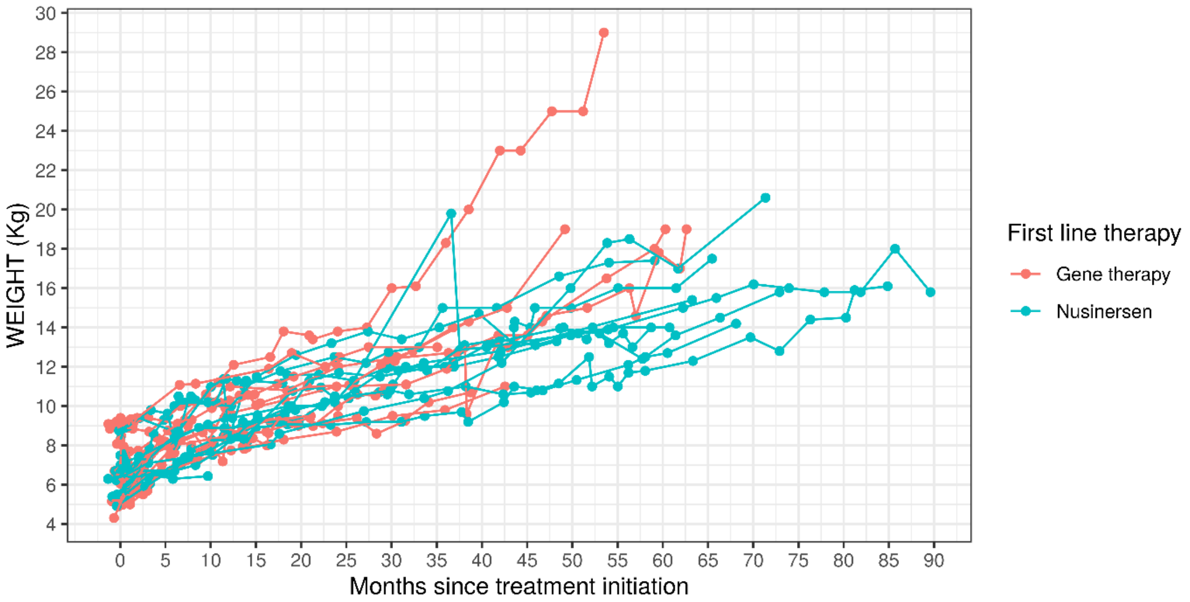


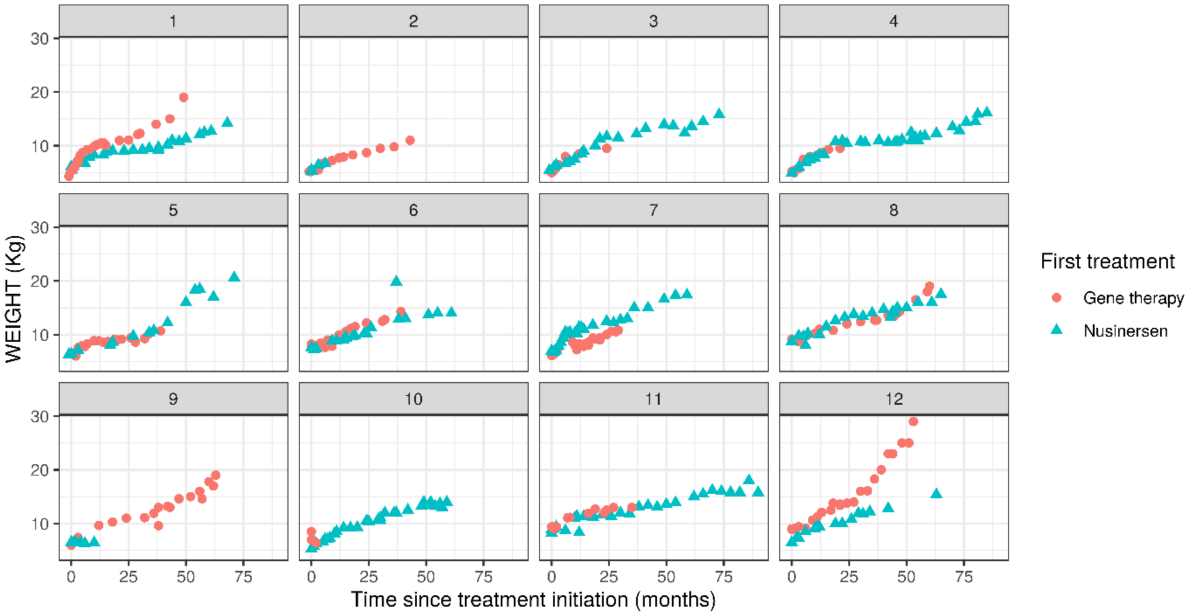


© 2025 Ropars J et al. *JAMA Network Open*.

# eTable 3: Individual-level data used to generate Kaplan-Meier ventilation-free and nutrition support–free survival curves

## eTable 3A: Individual-level data used to generate the Kaplan-Meier Ventilation-free survival curves

| Patient ID | Pair ID | Treatment | Time to event or censoring (months) | Event Status | Event Type |
|---|---|---|---|---|---|
| 1 | 1 | NsN | 41.63 | Event | Ventilation |
| 2 | 1 | GTx | 17.97 | Event | Ventilation |
| 3 | 2 | GTx | 42.54 | Censored | Censored |
| 4 | 2 | NsN | 1.08 | Event | Ventilation |
| 5 | 3 | GTx | 0.03 | Event | Ventilation |
| 6 | 3 | NsN | 8.25 | Event | Ventilation |
| 7 | 4 | GTx | 0.20 | Event | Ventilation |
| 8 | 4 | NsN | 4.47 | Event | Ventilation |
| 9 | 5 | GTx | 0.92 | Event | Ventilation |
| 10 | 5 | NsN | 14.49 | Event | Ventilation |
| 11 | 6 | GTx | 1.25 | Event | Ventilation |
| 12 | 6 | NsN | 0.95 | Event | Ventilation |
| 13 | 7 | GTx | 11.73 | Event | Ventilation |
| 14 | 7 | NsN | 0.82 | Event | Ventilation |
| 15 | 8 | GTx | 60.29 | Censored | Censored |
| 16 | 8 | NsN | 5.22 | Event | Ventilation |
| 17 | 9 | GTx | 38.24 | Event | Ventilation |
| 18 | 9 | NsN | 2.00 | Event | Ventilation |
| 19 | 10 | NsN | 2.33 | Event | Ventilation |
| 20 | 10 | GTx | 1.94 | Event | Death |
| 21 | 11 | NsN | 15.90 | Event | Ventilation |
| 22 | 11 | GTx | 35.06 | Censored | Censored |
| 23 | 12 | GTx | 53.49 | Censored | Censored |
| 24 | 12 | NsN | 38.83 | Event | Ventilation |

© 2025 Ropars J et al. *JAMA Network Open*.

## eTable 3B Individual-level data used to generate the Kaplan-Meier Nutrition support-free survival curves

| Patient ID | Pair ID | Treatment | Time to event or censoring (months) | Event Status | Event Type |
|---|---|---|---|---|---|
| **1** | 1 | NsN | 41.66 | Event | Feeding support |
| **2** | 1 | GTx | 51.55 | Censored | Censored |
| **3** | 2 | GTx | 42.54 | Censored | Censored |
| **4** | 2 | NsN | 4.60 | Event | Feeding support |
| **5** | 3 | GTx | 24.11 | Censored | Censored |
| **6** | 3 | NsN | 10.19 | Event | Feeding support |
| **7** | 5 | GTx | 43.53 | Censored | Censored |
| **8** | 5 | NsN | 36.14 | Event | Feeding support |
| **9** | 6 | GTx | 42.87 | Censored | Censored |
| **10** | 6 | NsN | 1.35 | Event | Feeding support |
| **11** | 7 | GTx | 30.55 | Censored | Censored |
| **12** | 7 | NsN | 0.82 | Event | Feeding support |
| **13** | 8 | GTx | 60.29 | Censored | Censored |
| **14** | 8 | NsN | 69.52 | Censored | Censored |
| **15** | 9 | GTx | 62.62 | Censored | Censored |
| **16** | 9 | NsN | 10.18 | Event | Death |
| **17** | 10 | NsN | 3.12 | Event | Feeding support |
| **18** | 10 | GTx | 1.94 | Event | Death |
| **19** | 11 | NsN | 89.59 | Censored | Censored |
| **20** | 11 | GTx | 35.06 | Censored | Censored |
| **21** | 12 | GTx | 53.49 | Censored | Censored |
| **22** | 12 | NsN | 77.60 | Censored | Censored |

© 2025 Ropars J et al. *JAMA Network Open*.

## eTable 4: Maximal motor abilities over time in matched patients according to the first-line therapy

| Variables | Matched Gene Therapy population N=12 | Matched nusinersen population N=12 |
|---|---|---|
| Maximal motor ability at treatment initiation (N=) | 12 | 12 |
| No capacity (%) | 5 (41.7) | 4 (33.3) |
| Head control (more than 3sec) (%) | 6 (50.0) | 7 (58.3) |
| Sitting with support (%) | 1 (8.3) | 1 (8.3) |
| Age at Maximal motor ability at treatment initiation (months) | 12 | 12 |
| mean(std) | 5.9(3) | 5.9(3) |
| med[IQ] | 4.5[4.1;8.4] | 4.7[3.5;7.7] |
| min-max | 1.6-11.4 | 2.8-11.1 |
| Maximal motor ability at 1 year post first treatment initiation (N=) | 12 | 12 |
| No capacity (%) | 0 (0.0) | 1 (8.3) |
| Head control (more than 3sec) (%) | 5 (41.7) | 3 (25.0) |
| Sitting with support (%) | 3 (25.0) | 3 (25.0) |
| Sitting without support (%) | 3 (25.0) | 4 (33.3) |
| Deceased (%) | 1 (8.3) | 1 (8.3) |
| Maximal motor ability at 2 years post first treatment initiation (N=) | 12 | 12 |
| Head control (more than 3sec) (%) | 0 (0.0) | 1 (8.3) |
| Sitting with support (%) | 2 (16.7) | 3 (25.0) |
| Sitting without support (%) | 8 (66.7) | 5 (41.7) |
| Standing with support (%) | 1 (8.3) | 1 (8.3) |
| Deceased (%) | 1 ( 8.3) | 2 (16.7) |
| Maximal motor ability at 3 years post first treatment initiation* (N=) | 9 | 12 |
| Sitting with support (%) | 0 (0.0) | 1 (8.3) |
| Sitting without support (%) | 6 (66.7) | 5 (41.7) |
| Standing with support (%) | 1 (11.1) | 4 (33.3) |
| Walking with support (%) | 1 (11.1) | 0 ( 0.0) |
| Deceased (%) | 1 (11.1) | 2 (16.7) |
| Maximal motor ability at 4 years post first treatment initiation* (N=) | 5 | 12 |
| Sitting with support (%) | 0 (0.0) | 1 (8.3) |
| Sitting without support (%) | 2 (40.0) | 4 (33.3) |
| Standing with support (%) | 1 (20.0) | 4 (33.3) |
| Standing without support (60sec) (%) | 0 (0.0) | 1 (8.3) |
| Walking with support (%) | 1 (20.0) | 0 ( 0.0) |
| Deceased (%) | 1 (20.0) | 2 (16.7) |
| Maximal motor ability at 5 years post first treatment initiation* (N=) | 3 | 12 |
| Sitting with support (%) | 0 (0.0) | 1 (8.3) |
| Sitting without support (%) | 1 (33.3) | 4 (33.3) |
| Standing with support (%) | 1 (33.3) | 3 (25.0) |
| Standing without support(60sec) (%) | 0 (0.0) | 1 (8.3) |
| Walking with support (%) | 0 (0.0) | 1 (8.3) |
| Deceased (%) | 1 (33.3) | 2 (16.7) |

© 2025 Ropars J et al. *JAMA Network Open*.

| **Variables** | **Matched Gene Therapy population N=12** | **Matched nusinersen population N=12** |
|---|---|---|
| Maximal miotor ability at 6 years post first treatment initiation* (N=) | 1 | 7 |
| Sitting without support (%) | 0 ( 0.0) | 1 (14.3) |
| Standing with support (%) | 0 ( 0.0) | 4 (57.1) |
| Deceased (%) | 1 (100.0) | 2 ( 28.6) |
| Maximal motor ability at 7 years post first treatment initiation* (N=) | 1 | 4 |
| Sitting without support (%) | 0 ( 0.0) | 1 (25.0) |
| Standing with support (%) | 0 ( 0.0) | 1 (25.0) |
| Deceased (%) | 1 (100.0) | 2 ( 50.0) |

© 2025 Ropars J et al. *JAMA Network Open*.

# eTable 5: Unsatisfactory clinical response (UCR)

Conditional logistic regression

Association between UCR and first line treatment

| First line treatment | UCR | Crude matched Odds Ratio [95% CI][a] |
|---|---|---|
| GTx N(%) | 3 (25) | 1 |
| Nusinersen N(%) | 8 (66.7) | 2.667 [0.7075 ; 10.05] |

[a]: Conditional logistic regression model

CI: confidence interval

Exact McNemar's test

To account for the small sample size, we computed an exact McNemar test.

Results when GTx is the reference : OR = 5.9999   95% IC [0.7279 ; 275.6384]  p value = 0.125

Results when Nusinersen is the reference : OR = 0.16667  95% IC [0.0036 ; 1.3737]   p value = 0.125

© 2025 Ropars J et al. *JAMA Network Open*.
