# Supplementary material for: Comparative Clinical Outcomes of Nusinersen and Gene Therapy in Spinal Muscular Atrophy Type 1
Source: JAMA Netw Open. 2025 Oct 8;8(10):e2536348. doi: 10.1001/jamanetworkopen.2025.36348 (PMC12508997; doi:10.1001/jamanetworkopen.2025.36348)
Supplement: Supplement 2. — Members of the French SMA Registry Study Group [file jamanetwopen-e2536348-s002.pdf]

Supplemental Online Content: Nonauthor Collaborators
*First name, last name, and suffix (if applicable) are required and will appear in PubMed.

***Group Name(s): French SMA Registry Study Group**

*First Name and Middle Initial(s)	*Last Name	*Suffix (eg, Jr, III)	Academic Degrees	Institution	Location (city, state/province, country)	Role or Contribution, eg, chair, principal investigator	Group (if more than 1 Group listed in the byline) and/or Subgroup (eg, Steering Committee)
Djillali	Annane		MD, PHD	AP-HP Garches (RPC) - Réanimation	Garches	principal investigator	
Shahram	Attarian		MD, PHD	AP-HM Marseille - Adultes	Marseille	principal investigator	
Nathalie	Bach		MD	CHU-Caen Adultes	Caen	principal investigator	
Rémi	Bellance		MD	CHU-Martinique - Adultes	Martinique	principal investigator	
Patrick	Berquin		MD, PHD	CHU-Amiens - Pédiatrie	Amiens	principal investigator	
Pierre	Beze-Beyrie		MD	CH- Pau - Pédiatrie	Pau	principal investigator	
Francois Constant	Boyer		MD, PHD	CHU Reims Adultes	Reims	principal investigator	
Brigitte	Chabrol		MD, PHD	AP-HM Marseille - Pédiatrie	Marseille	principal investigator	
Mondher	Chouchane		MD	CHU- Dijon -Pediatrie - Adultes	Dijon	principal investigator	
Ariane	Choumert		MD	CHU- Reunion Saint Pierre - Adultes	Saint Pierre	principal investigator	
Pascal	Cintas		MD	CHU-Toulouse - Adultes	Toulouse	principal investigator	
Elisa	De La Cruz		MD	CHU-Montpellier - Adulte	Montpellier	principal investigator	
Kumaran	Deiva		MD, PHD	AP-HP Bicêtre (BCT) - Pédiatrie	Le Kremlin-Bicêtre	principal investigator	
Klaus	Dieterich		MD	CHU- Grenoble - Pédiatrie	Grenoble	principal investigator	
Sophie	Duclos		MD	CHU- Martinique - Adultes	Martinique	principal investigator	
Andoni	Echaniz-Laguna		MD	AP-HP Bicêtre (BCT) - Adultes	Le Kremlin-Bicêtre	principal investigator	
Narcisse	Elenga		MD, PHD	CH-Cayenne Guyane - Pédiatrie	Guyane	principal investigator	
Caroline	Espil-Taris		MD	CHU- Bordeaux -Pédiatrie	Bordeaux	principal investigator	
Olivier	Flabeau		MD	CH- Cote Basque Bayonne - Adultes	Bayonne	principal investigator	
Melanie	Fradin		MD	CHU- Rennes - Pédiatrie et adultes	Rennes	principal investigator	
Rachel	Froget		MD	CHU- Limoges - Pédiatrie	Limoges	principal investigator	
Karima	Ghorab		MD	CHU- Limoges - Adultes	Limoges	principal investigator	
Gaëlle	Gousse		MD	CHU- Saint-Etienne - Pédiatrie	Saint-Etienne	principal investigator	
Marine	Guichard		MD	CHU- Tours - Pédiatrie	Tours	principal investigator	
Lucie	Guyant-Marechal		MD	CHU- Rouen - Adultes	Rouen	principal investigator	
Agnès	Jacquin-Piques		MD, PHD	CHU- Dijon -Pediatrie - Adulte	Dijon	principal investigator	
Anne-Laure	Kaminsky		MD	CHU- Saint-Etienne - Adultes	Saint-Etienne	principal investigator	
Pascal	Laforet		MD, PHD	AP-HP Garches (RPC) - Adultes	Garches	principal investigator	
Clémentine	Lambert		MD	CHRU- Nancy - Pédiatrie	Nancy	principal investigator	

*First Name and Middle Initial(s)	*Last Name	*Suffix (eg, Jr, III)	Academic Degrees	Institution	Location (city, state/province, country)	Role or Contribution, eg, chair, principal investigator	Group (if more than 1 Group listed in the byline) and/or Subgroup (eg, Steering Committee)
Leila	Lazaro		MD	CH- Cote basque Bayonne - Pédiatrie	Bayonne	principal investigator	
Edoardo	Malfatti		MD, PHD	HMN - Créteil - Adultes	Créteil	principal investigator	
Sandra	Mercier		MD, PHD	CHU de Nantes--Adultes	Nantes	principal investigator	
Philippe Edouard	Merle		MD	CHU- Amiens - Adulte	Amiens	principal investigator	
Maud	Michaud		MD	CHRU- Nancy - Adultes	Nancy	principal investigator	
Aleksandra	Nadaj-Pakleza		MD	CHRU- Strasbourg - Adultes	Strasbourg	principal investigator	
Sylvain	Nollet		MD	CHU- Besançon - Adultes	Besancon	principal investigator	
Marie-Christine	Nouges		MD	AP-HP Trousseau	Paris	investigator	
Jean-Baptiste	NOURY		MD	CHU- Brest - Adultes	Brest	principal investigator	
Yann	Pereon		MD, PHD	Nantes-Pédiatrie	Nantes	principal investigator	
Anne	Pervillé		MD	Saint-Pierre - Pédiatrie2	Saint-Pierre	principal investigator	
Christian	Richelme		MD	HPU- Lenval Nice - Pédiatrie	Nice	principal investigator	
Pascal	Sabouraud		MD	CHU- Reims - Pédiatrie	Reims	principal investigator	
Sabrina	Sacconi		MD, PHD	CHU- Nice - Adultes	Nice	principal investigator	
Elisabeth	Sarrazin		MD	CHU- Martinique - Pédiatrie	Martinique	principal investigator	
Catherine	Sarret		MD, PHD	CHU- Clermont-Ferrand Pédiatrie	Clermont-Ferrand	principal investigator	
Cyril	Schweitzer		MD, PHD	CHRU- Nancy - Pédiatrie	Nancy	principal investigator	
Guilhem	Sole		MD	CHU- Bordeaux - adultes	Bordeaux	principal investigator	
Marco	Spinazzi		MD	CHU d'Angers - Adultes	Angers	principal investigator	
Tanya	Stojkovic		MD, PHD	AP-HP Paris (PSL) - Adultes	Paris	principal investigator	
Marie	Thibaud		MD	CHU- Reims - Pédiatrie	Reims	principal investigator	
Valérie	Trommsdorf		MD	CHU- Reunion Saint Pierre - Pédiatrie	Saint Pierre	principal investigator	
Jon Andoni	Urtizberea		MD	Institut de Myologie Paris	Paris	principal investigator	
Catherine	Vanhulle		MD	CHU- Rouen - Pédiatrie	Rouen	principal investigator	
Carole	Vuillerot		MD, PHD	CHU- Lyon - Pédiatrie et Adultes	Lyon	principal investigator	
