# Supplementary material for: Comparative Clinical Outcomes of Nusinersen and Gene Therapy in Spinal Muscular Atrophy Type 1
Source: JAMA Netw Open. 2025 Oct 8;8(10):e2536348. doi: 10.1001/jamanetworkopen.2025.36348 (PMC12508997; doi:10.1001/jamanetworkopen.2025.36348)
Supplement: Supplement 3. — Data Sharing Statement [file jamanetwopen-e2536348-s003.pdf]

Data Sharing Statement

Ropars. Comparative Clinical Outcomes of Nusinersen and Gene Therapy in Spinal Muscular Atrophy Type 1. *JAMA Netw Open*. Published October 08, 2025.
doi:10.1001/jamanetworkopen.2025.36348

Data
Data available: Yes
Data types: Deidentified participant data
How to access data: All data included in this analysis are recorded in the French SMA registry. Data can be obtained anonymized and aggregated upon request and approval by the French SMA registry committee. The study protocol is available on supplementary data.
When available: With publication

Supporting Documents
Document types: Informed consent form, Statistical/analytic code
How to access documents: Data can be obtained anonymized and aggregated upon request and approval by the French SMA registry committee. Please contact the corresponding author at juliette.ropars@chu-brest.fr
When available: With publication

Additional Information
Who can access the data: researchers whose proposed use of the data has been approved by the French SMA registry committee
Types of analyses: for a specified purpose
Mechanisms of data availability: after approval of a proposal (with a signed data access agreement)
